# Epidemiology of myocardial injury in trauma patients: proposed phenotypes for future research

**DOI:** 10.1007/s00068-025-02798-7

**Published:** 2025-02-28

**Authors:** Jett Karolewski, Jodie-Kate Williams, Natasha Weaver, Simone Meakes, Karen Gane, Zsolt J. Balogh

**Affiliations:** 1 Department of Traumatology, John Hunter Hospital, Newcastle, NSW Australia; 2 Injury & Trauma Research Program, Hunter Medical Research Institute, Newcastle, NSW Australia; 3 Discipline of Surgery, School of Medicine and Public Health, University of Newcastle, Newcastle, NSW Australia

## Abstract

**Purpose:**

To describe the epidemiology of myocardial injury in trauma patients, in doing so informing design for future multicentre prospective studies.

**Method:**

A one-year retrospective study ending on 31/08/2023 was conducted at a Level-1 Trauma Centre. All adult trauma resuscitation patients with elevated Troponin serum concentration were included. Patient demographics, medical history, mechanism, injury severity, laboratory data, cardiac investigations, LOS, ICU admission and mortality were collected. Patients were categorised into three pragmatic groups based on the timing of their Troponin peak (Group1:<12 h; Group2:12–24 h; Group3:>24 h).

**Results:**

From 1408 admissions, 97(7%) patients [Age:57(35,80); Male:71%; ISS:18(9–33); LOS:9(4,16.5); ICU:66%; Mortality:16.5%] had elevated Troponin. Group 1 [*n* = 37; Age:47(24,70); Male:76%; ISS:9(4,22); LOS:7(3,14); ICU:51%; Mortaliy:5.4%]; Group 2 [*n* = 32; Age:53.5(26,74); Male:78%; ISS:27(12.5,53.5); LOS:10(5,17); ICU:84%; Mortaliy:25%] and Group 3 [*n* = 28; Age:78(62,84); Male:57%; ISS:19(9.5,47.5); LOS:12.5(6,19.5); ICU:64%; Mortaliy:21%]. 64% of patients had thoracic injuries, which was consistent among the three groups. Group 3 had most frequent ECG (61%) and echocardiography (25%) findings.

**Conclusion:**

Troponin elevation occurs in 7% of all trauma admissions and it identifies the seriously injured high-risk cohort. The timing of the maximum Troponin concentration seems to describe three distinct phenotypes. “Hyperacute” with most favourable outcomes, “Subacute” with severe trauma and tissue injury requiring major resource utilisation and associated with the highest mortality rate, and “Late” characterised by ECG and ECHO changes suggesting primary ischaemic cardiac pathology.

## Background

Myocardial injury is a potentially morbid and lethal condition in the trauma population [1]. It can be due to direct trauma to the heart (blunt cardiac injury), peri-traumatic ischaemic cardiac event or systemic inflammation causing secondary myocardial injury. Direct trauma can result in pathologies such as myocardial rupture, contusion, septal tears, valvular injury, arrhythmias, and coronary artery thrombosis or laceration. The true incidence of myocardial injury in trauma patients is unknown and varies greatly in the literature due to the variability in definition, examined population and the potential paucity of the aforementioned aetiologies. Additionally, myocardial injury detection in trauma patients is particularly challenging due to a lack of diagnostic standardisation.

Troponin serum concentration is a sensitive and specific marker of myocardial cell damage currently in widespread use [2]. The Fourth Universal Definition of Myocardial Infarction incorporates Troponin result, which must be above the 99th percentile upper reference limit. In defining a myocardial infarct, a patient must have a raised Troponin result as well as either symptoms of myocardial ischaemia, new ECG changes or imaging consistent with ischaemic pathology. A type 2 myocardial infarct has the same definition however is due to an imbalance between myocardial oxygen supply and demand unrelated to coronary thrombosis. The interpretation of Troponin result goes hand in hand with diagnosis of myocardial injury [3]. The measurement of Troponin in a trauma patient can detect myocardial injury regardless of the aetiology with some variation in sensitivity and specificity due to the frequently associated major tissue injury. In critically injured patients, a higher Troponin concentration has been demonstrated to be associated with more frequent cardiac and non-cardiac complications, as well as with higher mortality rates [4].

There is limited information available on the timing and natural history of elevated Troponin concentration applicable to the population including all trauma admissions. Being better informed in this field could assist with characterisation, management and outcome prediction of major trauma patients.

This study aims to describe the epidemiology and natural history of myocardial injury defined by elevated Troponin concentrations in trauma patients to inform power calculation and design for future multicentre prospective studies.

## Methodology

A one-year retrospective review was conducted in a Level 1 Trauma Centre. The Trauma Registry was accessed to identify all trauma admissions between 1st September 2022–31st August 2023. These patients were then screened and included if they had an elevated Troponin result during their admission. Troponin I was the Troponin assay used in this study as it is the standard in the State of New South Wales. An elevated Troponin was defined as greater than or equal to 26ng/L as per the local Pathology recommended cut-off. For pragmatic purposes, it was assumed that if Troponin was not measured, the clinical suspicion was negligible and the patient did not have clinically relevant cardiac injury. Inclusion criteria were patients older than 16 years of age and admitted by the trauma service via trauma team activation. Patients with all injury severity were included.

Patients were categorised into three groups based on the timing of their peak Troponin concentration after injury (Group 1: <12 h; Group 2: 12–24 h; Group 3: >24 h). The data collection from the prospectively maintained trauma registry and focused review of the electronic medical records included patient demographics, medical history, mechanism of injury, injury severity, laboratory data, cardiac investigations, length of stay, admission to the Intensive Care Unit and mortality. The causes of mortality were obtained via the multidisciplinary death review panel data. Data is presented as percentages, mean and standard deviation or median and interquartile range. P-value is from an overall test of difference between the 3 groups. For categorical variables, Fisher’s exact test was used. For continuous variables, Kruskal-Wallis test was used.

### Ethics approval

for this project was obtained from the Hunter New England Research Ethics Committee in 2023 (Approval number: AU202308-03). No potential conflict of interest has been identified.

## Results

**Fig. 1:**
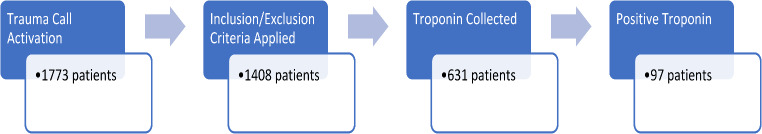
Flow chart demonstrating patient selection with application of inclusion and exclusion criteria

During the 12-month study period 1773 trauma patients were admitted under the Trauma Service through the trauma team activation system. After inclusion and exclusion criteria was applied, 1408 patients were considered for this study. Of these patients, 631 (45%) had a Troponin measurement during their admission and 97 patients (7%) had a positive Troponin result during their admission (See Fig. 1).

**Fig. 2:**
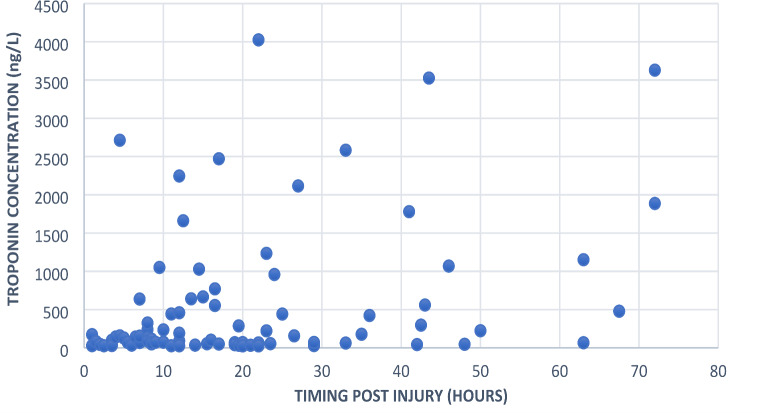
Scatterplot graph illustrating distribution of peak Troponin concentration results by timing post injury for all patients with positive Troponin concentration during admission

The below scatterplot graph demonstrates the distribution of peak Troponin concentration result by the number of hours this result occurred post injury. The majority of these peak results (71%) occurred within the first 24 h post injury, and 43% of all patients had only mild Troponin elevation of less than 100ng/L (See Fig. 2).

Of the 97 patients with a positive Troponin concentration during their admission, 71% were male and the mean age was 57 (IQR 35–80) years. 69% of patients had thoracic injuries and the median Injury Severity Score (ISS) was 18 (IQR 9–33). Median length of stay was 9 (IQR 4-16.5) days. 66% of patients required Intensive Care Unit (ICU) admission and the mortality rate was 16.5%. The mean eGFR was 68mL/min/1.73m^2^ ± 24 and the mean Creatinine was 111umol/L ± 69. 37% of patients received catecholamines during their admission and 15 patients (42% of this subgroup) received catecholamines prior to their peak Troponin result. 13.5% had positive findings on echocardiogram and 35% were documented to have ECG changes. Positive ECG changes include tachycardia not explained by haemodynamic status, new onset of atrial fibrillation, ST or T wave changes, bundle branch block, bradycardia, bigeminy and ectopic beats. Positive echocardiogram findings include pericardial effusion, hypokinesis, valvular pathologies and dilated heart chambers. 13.5% of patients were recommended to have formal Cardiology follow-up on discharge. Some reasons for this included new atrial fibrillation, myocardial infarct, heart failure optimisation and patients who had a primary cardiac event as the precipitant of their injury.

Patients were divided into three groups based on the timing of peak Troponin release post injury. The results are shown in Table 1. The highest mortality rate, ICU admission rate and ISS were seen in the subacute group (peak Troponin concentration 12–24 h post injury). The late group (peak Troponin concentration > 24 h post injury) demonstrated higher rates of positive ECG and echocardiogram findings.

**Table 1:** Data collected for each group of timing of peak troponin release post injury. LOS, ISS and Age are presented as median (IQR). Creatinine and eGFR are presented as mean ± standard deviation

	PEAK TROPONIN TIME POST INJURY			
*Variable*	**HYPERACUTE** **< 12 h** **(n = 37)**	**SUBACUTE** **12–24 h** **(n = 32)**	**LATE** **> 24 h** **(n = 28)**	**P-value**
*Male*	28 (76%)	25 (78%)	16 (57%)	0.174
*Age*	47 (24, 70)	53.5 (26, 74)	78 (62, 84)	< 0.001
*Thoracic injury*	24 (65%)	23 (72%)	19 (68%)	0.825
*ISS*	9 (4, 22)	27 (12.5, 53.5)	19 (9.5, 47.5)	0.003
*LOS (days)*	7 (3, 14)	10 (5, 17)	12.5 (6, 19.5)	0.108
*ICU admission*	19 (51%)	27 (84%)	18 (64%)	0.013
*Mortality rate*	2 (5.4%)	8 (25%)	6 (21%)	0.050
*Positive * *ECG findings*	9 (24%)	8 (25%)	17 (61%)	0.005
*Positive * *ECHO findings*	2 (5.4%)	4 (13%)	7 (25%)	0.078
*Creatinine*	116 ± 92	105 ± 43	112 ± 58	0.534
*eGFR*	72 ± 23	71 ± 21	60 ± 27	0.058

Relevant comorbidities among the three cohorts include hypertension, dyslipidaemia, diabetes, renal failure, ischaemic heart disease, atrial fibrillation and heart failure. In the hyperacute cohort 40.5% of patients had comorbidities, then 37.5% of the subacute cohort and 78.6% of the late cohort.

There were 16 mortality cases in total. 10 were due to predominantly central nervous system related injuries including to brain, brainstem and high c-spine, two patients died from the consequences of multiple organ failure and one patient due to type 2 respiratory failure. 7 patients in our study underwent CPR and were included in analysis.

## Discussion

Our results showed that pathological Troponin concentration elevation occurs in 7% of all trauma patients. According to the New South Wales (NSW) Trauma Registry [5], there were a total of 3793 adult trauma service admissions in NSW from 2020 to 2021. Therefore, across the state approximately five trauma service admissions every week will have an elevated Troponin suggestive of myocardial injury. The incidence of Troponin elevation in trauma patients varies greatly in the literature, with some studies reporting rates of 12%, 42% and 66% [6–8]. These studies have a lower threshold for positive Troponin result (> 14ng/L) as well as stricter inclusion criteria including ISS > 15 or 16, or patients admitted to ICU only. These factors likely contribute to the higher proportion of positive Troponin results in these studies when compared to our study. These studies showed that Troponin elevation is associated with significantly poorer adjusted outcomes, particularly with higher mortality rate [7, 8]. The mortality in our cohort is also much higher than is expected from the overall ISS (median 18), indicating that elevated Troponin is a surrogate marker of poor outcomes, which confirms the findings of other studies [7, 8].

In our study three distinct groups are described based on timing of maximum Troponin concentration– “Hyperacute”, “Subacute” and “Late”. The “hyperacute” troponin peak (within 12 h) was associated with the mildest injury and lowest mortality rate. Patients with a “subacute” peak Troponin concentration had the highest ISS, higher mortality rates and were more likely to require ICU admission. These patients are reflective of the consequences of severe injury with traumatic shock and tissue injury. Patients with a “late” rise in Troponin concentration were more than twice as likely to have ECG and echocardiography changes. This shows that there is an increased incidence of structural and conductive cardiac pathology in those patients with a peak troponin > 24 h post injury, when compared with the other subgroups.

All groups in our study demonstrated high prevalence of thoracic injuries, highlighting the strong correlation between blunt thoracic trauma and myocardial injury. Patients with significant thoracic trauma have a 13% chance of blunt myocardial contusion [9] and thoracic trauma has been shown to be strongly associated with Troponin elevation [10].

Of our participants, 43% were elderly (65 and older) and one third of these elderly patients had an operation during their admission. It is important to note the higher risk of perioperative cardiac complications in the elderly population. These complications include acute coronary syndrome, heart failure, new arrhythmias and cardiac arrest. These can play a critical role in the incidence of myocardial injury in this population.

Secondary myocardial injury without direct cardiac injury is predominantly seen in polytrauma patients admitted to the ICU in the context of traumatic shock, severe inflammatory response, organ dysfunction and multiple organ failure. Factors that contribute to this include adrenergic stress, hypovolaemic shock and reperfusion injury, production of bacterial myocardial toxins, use of cardio-active inotropes, and microcirculatory dysfunction [2]. Given the size and exploratory nature of our study we cannot comment confidently on this association but certainly our data suggests the validity of this hypothesis, which can be explored based on our findings, which are able to inform power calculation for future prospective studies.

Only one other study has been found that investigates the timing of post injury peak Troponin release [6]. Similarly to our study, Riou divided patients into three separate groups. The first included patients who had a limited Troponin release (defined as < 2000ng/L) within 12 h of injury. The second group had a significant Troponin release (≥ 2000ng/L) less than 36 h post injury. And the third group had a sustained and significant Troponin release more than 36 h post injury. It was found that patients with a more acute Troponin release had myocardial injury related to the hyperadrenergic state observed in haemorrhagic shock or severe injury. All patients who met the criteria for delayed and significant Troponin release underwent coronary artery angiography and results showed that 47% of these patients had a coronary artery injury such as dissection or occlusion. This supports our study results - that patients with a delayed Troponin rise had findings suggestive of a primary ischaemic cardiac pathology.

The timing of Troponin release post injury can be used as a tool to help guide screening and prognosis of trauma patients. Routine Troponin checks for all trauma patients and a serial check in the case of abnormality could be beneficial as a prognostic tool in trauma patients, and this could be further investigated in future studies. We propose that in patients with Troponin peaking within 12 h, myocardial injury can be a self-limiting process and may not require further monitoring, diagnostics and intervention.

There are some limitations to this study. We used serum Troponin as a screening tool for myocardial injury, in doing so assuming that Troponin measurements were performed in any trauma patients with clinical concern for cardiac injury. Future prospective studies could screen all trauma resuscitation patients with a Troponin concentration for consistency and reliability of results. This study had a sample size of 97 patients. A larger scale study would be useful to ensure the statistical significance of results. Additionally, this study solely investigates trauma patients with raised Troponin concentration. A future study could be conducted to compare variables between Trauma admissions who had elevated Troponin concentration and those who did not. This comparison could provide data on the difference between presentation, prognosis and management of these two cohorts.

A further limitation of our study is that our Cardiology service has a non-standardised approach to reporting echocardiograms and in addition to this the reporting is generally not specific to blunt cardiac injury. The same is applicable to our radiology reporting, and conditions relevant to blunt cardiac injury, for example *contusion cordis*, are not reported on CT scans. We have included in our study the relevant echocardiogram results in relation to our cohort, however our study was not designed or powered to assess specific echocardiogram findings in blunt cardiac injury. Similarly, although we mention relevant co-morbidities in our study results, our study was not powered to identify potential risk factors in blunt cardiac injury. Our study aim was to provide information on the epidemiology of myocardial injury and in doing so comment on the incidence of various factors in our identified cohort. Future studies that power these specific factors would be beneficial in exploring and analysing their relationship to blunt cardiac injury in more detail.

## Conclusion

Pathological Troponin concentration elevation occurs in 7% of all trauma patients and it identifies the seriously injured high-risk cohort with worse outcomes than expected based on injury severity. The timing of the maximum Troponin concentration seems to describe three distinct phenotypes. “Hyperacute”, which is seemingly a self-limiting process with most favourable outcomes; “Subacute” with severe trauma and tissue injury requiring major resource utilisation and associated with the highest mortality rate; and “Late” characterised by ECG and ECHO changes suggesting primary ischaemic cardiac pathology. This descriptive study provides essential information for future prospective studies investigating the significance of myocardial injury in the trauma population. We suggest further characterisation of the proposed phenotypes with aims to individualise their management for optimal outcomes.

## Data Availability

No datasets were generated or analysed during the current study.
